# Liver-Intestinal Involvement in Graft Versus Host Disease in Pediatric Hematopoietic Stem Cell Transplantation Patients: Ten Years of Experience in 2 Centers of Latin America

**DOI:** 10.1097/PG9.0000000000000211

**Published:** 2022-07-25

**Authors:** Andrea Sepúlveda, Gustavo Tagliaferro, Gabriel Arancibia, Francisco Barriga, Verónica Busoni, Marina Orsi

**Affiliations:** From the * Department of Pediatric Gastroenterology and Nutrition, Pontificia Universidad Católica de Chile; † Department of Pediatric Gastroenterology and Hepatology, Hospital Italiano de Buenos Aires; ‡ Department of Pediatric Hematology and Oncology, Pontificia Universidad Católica.

**Keywords:** Graft-versus-host disease

## Abstract

To describe the behavior and characteristics of children with diagnosis of graft versus host disease (GVHD) with liver-intestinal involvement.

**Methods:**

Retrospective cohort study of pediatric patients with history of hematopoietic stem cell transplantation for diagnosis of GVHD with gastrointestinal (GI) or liver involvement, from 2 pediatric centers.

**Results:**

Between 2007 and 2017, 57 pediatric patients presented with liver or intestinal GVHD; 74% with GI GVHD, 11% with liver GVHD, and 15% with liver-intestinal involvement. Diarrhea (96%) and abdominal pain (55%) were the most frequent symptoms. Endoscopies were performed in 88%, and 35% required a second procedure to confirm diagnosis. Normal-appearing mucosa was observed in 17% of upper GI endoscopies and in 29% of colonoscopies. Endoscopic pathological findings were observed mainly in colon (62%). There was greater severity on colonoscopic classification in those with liver-intestinal compromise than in those with GI compromise only. Overall mortality was 26%.

**Conclusion:**

GI and liver GVHD diagnosis may present serious complications. GI involvement tends to manifest early, so it is appropriate to suspect it in the first days after transplantation, unlike liver involvement, which occurs late when other organs are involved. We did not observe a direct relationship between endoscopic and histological classification. Both GI and liver involvement in GVHD could predict greater target organ involvement.

**What Is Known**

- Graft versus host disease is a severe complication in stem cell transplantation patients.
- Liver involvement is not frequent but could be fatal.
- Nutritional deterioration is multifactorial.

**What Is New**

- Diarrhea and abdominal pain were the principal symptoms.
- There is no direct relationship between histological and endoscopic classification.
- Liver and intestinal disease could predict severe organ involvement.

**A**llogeneic hematopoietic stem cell transplantation (HSCT) is a widely used therapy for the treatment of malignant and nonmalignant diseases. Since its inception, >50 000 HSCTs have been performed worldwide, including Latin America (1–3).

Graft versus host disease (GVHD), first described in 1955 (4), is a severe complication, with an incidence of 35% to 50% (5). GVHD is due to an inflammatory response generated by the donor’s T cells on the organs of the recipient (3). After cutaneous involvement, the gastrointestinal (GI) tract and liver are the most frequently affected organs, associated with short- and long-term morbidity.

GI involvement usually presents with abdominal pain or persistent diarrhea, generally with greater involvement of the lower GI tract. The diagnosis is based on clinical, endoscopic, and histological features (6,7), always excluding other etiologies that can mimic this pathology. Liver involvement, less frequent following HSCT, can be suspected with acute elevation of bilirubin, alkaline phosphatase, and transaminases, requiring liver biopsy to confirm GVHD. It has been described in <5% of patients as a fatal complication, with no effective treatment (8).

A secondary complication to GVHD relates to the nutritional aspects, which is multifactorial, including lengthy hospital stay, persistent catabolic state after conditioning, and prolonged use of corticosteroids. While these findings are well described in adults, few cases are reported in the pediatric population, which is more vulnerable to nutritional deterioration.

Much of the available literature is based on observational studies in adults. This study describes the behavior and characteristics of GI-liver GVHD in 2 pediatric referral centers in Latin America. We describe the mode of presentation and management of the nutritional, digestive, and hepatic problems, analyzing clinical, laboratory, endoscopic, and histological features.

## MATERIALS AND METHODS

This retrospective observational descriptive study includes all patients with confirmed diagnosis of intestinal or hepatic GVHD who had undergone an HSCT for any diagnosis from January 2007 through January 2017. Recruitment sites were the Clinical Hospital of Universidad Católica de Chile and the Hospital Italiano de Buenos Aires, Argentina. The inclusion criteria were age at transplantation ≤18 years and upper endoscopy, colonoscopy, or GI or liver biopsy results confirming the diagnosis of GI or liver GVHD. In all cases, toxic and infectious causes were excluded.

The clinical presentation and the number of endoscopies of intestinal GVHD were recorded. The endoscopic and histological findings were described according to the Correa-Cruz/Friburgo and Lerner classifications, respectively (7,9). Hepatic GVHD was defined by clinical symptoms, laboratory, liver ultrasound, and histological confirmation.

The nutritional aspects of these patients were characterized according to the World Health Organization charts, using body mass index for those >2 years of age and weight/height index for those <2 years of age.

For all patients, general and clinical characteristics were described. Categorical variables, such as sex, presence of comorbidity, type of graft, type of donor, drugs, and type of involvement, are reported. Numerical variables, such as age at transplantation and number of transplants, with mean and SD or median and minimum and maximum are presented.

For the subgroup of patients with GI GVHD, the clinical, endoscopic, and histological presentation, the follow-up, and response to treatment were characterized using the number of cases and percentages for categorical variables and mean and SD or median and 25th percentiles for numerical variables. Additionally, for the combined distribution of patients who had both examinations, histological and endoscopic classifications were recorded. For the subgroup of patients with hepatic GVHD, the clinical, ultrasound, and histological presentation, follow-up, and response to treatment were characterized.

Nutritional status at diagnosis and follow-up was evaluated for all patients, and outcomes are described.

A bivariate analysis was performed for the scores obtained in the colonoscopic, histological, and upper endoscopic classifications. Scores were adjusted for sex, GVH involvement (intestinal only versus both intestinal and hepatic), presence of comorbidity (none versus at least one), and mortality using the Mann-Whitney *U* tests. Additionally, scores were compared according to graft type (bone marrow, umbilical cord blood, and peripheral blood) using the Kruskal-Wallis tests. Multiple comparison tests were performed using Bonferroni correction.

*P* value <0.05 level of significance was considered significant. Statistical analyses were performed with SPSS 17.0.

The protocol was approved by the ethics committee of both institutions with exemption of informed consent.

## RESULTS

Over the course of 10 years, 57 patients presented liver-GI GVHD in our hospitals: 33 in Chile and 24 in Argentina. Sixty-five percent were male with an age range between 0 and 17 (interquartile range, 9) years. Forty-five percent of the transplanted patients had acute lymphatic leukemia as diagnosis (Table 1).

**TABLE 1.** Cause of transplantation

Principal diagnosis	n (%)
Acute lymphatic leukemia	26 (45.6)
Myeloid leukemia	13 (22.8)
Myelodysplasia	4 (7.1)
Fanconi anemia	2 (3.5)
Hyper IgM syndrome	2 (3.5)
Medullary aplasia	2 (3.5)
Neuroblastoma	1 (1.8)
Wiskott-Aldrich syndrome	1 (1.8)
Non-Hodgkin lymphoma	1 (1.8)
Juvenile myelomonocytic leukemia	1 (1.8)
Severe combined immunodeficiency	1 (1.8)
Blackfan-Diamond anemia	1 (1.8)
Chronic granulomatous disease	1 (1.8)
α-Mannosidosis	1 (1.8)

Ninety percent had received 1 HSCT at the time of diagnosis, 5 patients had 2, and 1 had 3. The type of transplant was umbilical cord blood (40%), peripheral blood (32%), and bone marrow (28%), with histocompatible donors (72% unrelated donors). All received conditioning before transplantation according to the protocols of each institution; 61% total body irradiation associated with drugs. Of the 57, 74% developed GI GVHD, 11% liver, and 15% liver-GI. Other organs involved in these patients were skin, lungs, and eyes as shown in Table 2.

**TABLE 2.** Organ involved

Organ	n
Gastrointestinal tract	51
Skin	46
Liver	15
Eye	5
Lung	3

Regarding intestinal GVHD, 80% presented clinical compromise within the first 90 days after transplantation. Diarrhea (96%) and abdominal pain (55%) were the most frequent symptoms at the time of indicating endoscopy, followed by rectal bleeding (23%), vomiting (23%), and hematemesis (14%). All had biopsies obtained at the time of suspected diagnosis, 88% (45/51) of which were by digestive endoscopy. Half of these patients underwent both upper endoscopy and colonoscopy, and up to 35% required a second endoscopy to reach the diagnosis (double endoscopy in all cases, except 1 patient who underwent colonoscopy). In 6 patients, GVHD was diagnosed by rectal biopsy due to clinical instability.

**FIGURE 1.**
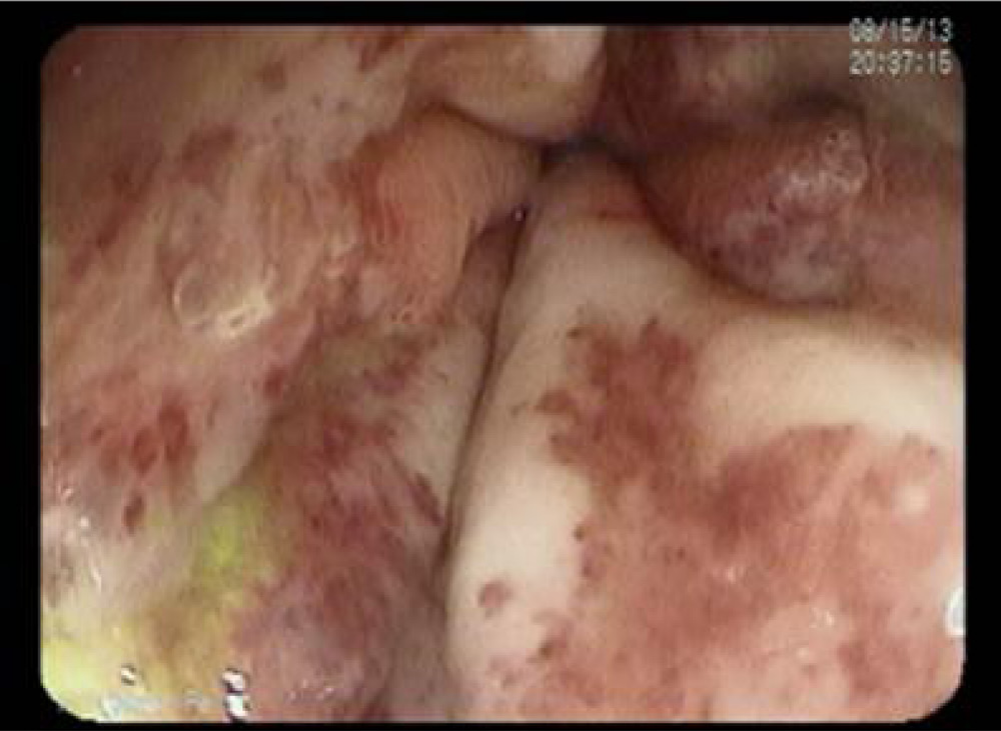
Esophageal graft versus host disease. Case 1: 16-year-old male patient. On day +80 of hematopoietic stem cell transplantation, an upper endoscopy was performed due to abdominal pain and hematemesis and revealed esophageal ulcerations with complete denudation of the mucosa.

**FIGURE 2.**
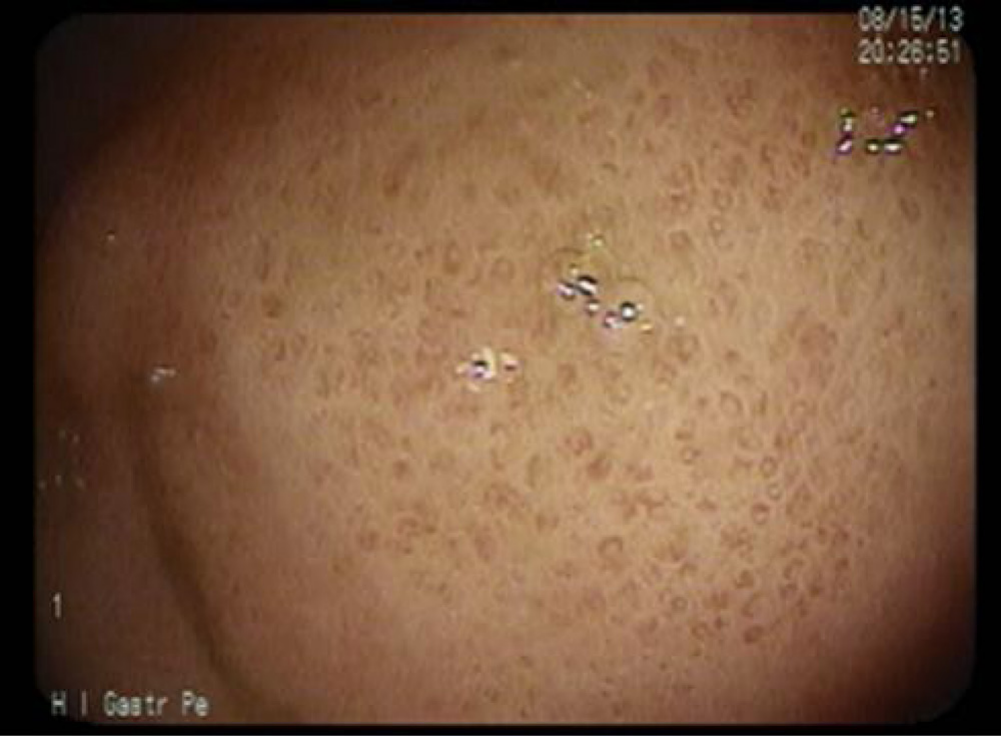
Gastric graft versus host disease. Case 1: stomach spotted erythema.

Upper endoscopic findings were normal in 17% of patients. Signs suggestive for esophagitis, gastropathy, and duodenopathy were found in 17%, 55%, and 45% of the cases, respectively (Fig. 1 and 2). Colonoscopy was normal in 29%, colitis (of variable extent) was found in 62%, and proctitis in 29%. Of the 7 that had an upper GI endoscopy histological classification equal to 4, 4 had a colonoscopic classification ≤1, 3 had a 2 or 3 classification, and none had a colonoscopy classification equal to 4.

All patients with liver involvement presented with variable elevation of serum transaminase and bilirubin levels, and only 30% manifested elevations during the first 90 days. Laboratory tests were performed as part of routine tests in asymptomatic patients and were performed in a targeted manner when patients presented with jaundice, anorexia, and abdominal pain. Hepatic involvement was suspected due to the persistent alteration in laboratory parameters (transaminase levels, serum bilirubin, gamma-glutamyl transferase, alkaline phosphatase). High levels of serum bilirubin were present in 25% of the patients, with numbers between 12 and 13 mg/dL. Doppler abdominal ultrasound at the time of suspected diagnosis was normal in most cases (11/14), highlighting only an increase in liver echogenicity in three.

Distribution by nutritional status was calculated according to the World Health Organization Child growth standards at the time of transplantation and GVHD diagnosis (Table 3). A decrease in weight/height or body mass index in *z* score was observed from a range of −2.8 to +3.5 at the time of transplantation to −3 to +2.7 at the time of GVHD. Twenty-four percent required enteral feeding by nasogastric tube, and 65% required parenteral nutrition during the course of their GVHD. The nutritional risk, acute malnutrition, and persistent low intake were the variables that led to the indication of parenteral nutrition, with a median duration of 30 days.

**TABLE 3.** Nutritional status

	Transplant, %	GVHD, %
Underweight	5.3	15
Risk weight	5.3	11
Normal weight	49.2	42
Overweight	21.1	24
Obesity	19.3	8

GVHD = graft versus host disease.

All patients received intravenous corticosteroids (1 to 2 mg/kg) as first-line therapy at the time of diagnosis, with almost all of them (52/57) requiring a second course or second-line therapy due to persistence of symptoms. The most frequently used drugs were cyclosporine, tacrolimus, infliximab, and mycophenolate.

More than one half (56%) of the patients had complications. Most were infectious (fungal infections, bacteremia, and viral infections), with a mortality of 26%. Septic shock and pneumonia were the complications associated with higher mortality. Two serious complications occurred as a result of the biopsy. One was an intramural hematoma of the duodenum after gastroscopy. In this case, conservative management including fasting for 45 days with parenteral nutrition for 2 months resolved the problem. The other was hypovolemic shock resulting from a transcutaneous liver biopsy puncture, leading to pseudoaneurysm in the right hepatic lobe on angiography requiring embolization.

When evaluating which modifiable or nonmodifiable variable could be related to severity, defined quantitatively by upper endoscopic, colonoscopic, and histological classification, no difference was found between sex or type of transplant. There was also no association with complications or mortality (Table 4); however, patients with GI and liver involvement presented with greater severity in the colonoscopic classification.

**TABLE 4.** Complication and mortality

	Complication			Mortality		
	Yes	No	*P* value	Yes	No	*P* value
Histology, n (%)			0.118			0.221
G1	8 (47.1)	9 (52.9)		2 (12.5)	14 (87.5)	
G2	8 (66.7)	4 (33.3)		3 (27.3)	8 (72.7)	
G3	6 (85.7)	1 (14.3)		3 (42.9)	4 (57.1)	
G4	5 (71.4)	2 (28.6)		2 (28.6)	5 (71.4)	
Colonoscopy, n (%)			0.394			0.367
G1	15 (57.7)	11 (42.3)		5 (20)	20 (80)	
G2	7 (63.6)	4 (36.4)		3 (30)	7 (70)	
G3	4 (100.0)	0 (0)		1 (25)	3 (75)	
G4	1 (50)	1 (50)		1 (50)	1 (50)	

There are 2 patients without information of mortality: one G1 and the other G2 (they did not return for medical consultation).

## DISCUSSION

Our retrospective cohort is the first report from South America that includes 2 hospitals with more than a decade of experience in HSCT. GI involvement was predominant, similar to reports in the literature (10).

In GI GVHD, the symptoms that were an indication for endoscopy were nonspecific and mostly in patients with multiple drug treatments, chemotherapy, and concomitant infections. We observed that 80% presented early, in the first 90 days, similar to that reported by Brodoefel et al (11). Abdominal pain and diarrhea were the most frequent symptoms at the time of clinical suspicion. Secretory diarrhea was common early post-transplant, being bloody in the following days or as the presentation of an advanced stage of GVHD. As the daily volume of watery stools was not quantified, it was not possible to classify according to the stages proposed by the International Registry of bone marrow trasplantation. In relation to our experience, we believe that this variable is not reproducible in pediatric clinical practice.

Currently, no consensus exists regarding the choice of the endoscopic procedure in children to confirm GI GVHD. In our cohort, the decision to perform an upper endoscopy, a colonoscopy, both, or a rectal biopsy was made based on the presenting symptom(s) and the clinical stability of the patient. Patients with diarrhea or proctorrhagia underwent colonoscopy. When vomiting or epigastric pain was present, a gastroscopy was performed. Upper endoscopy alone was performed in 4 patients, reserving the rectal biopsy for those with clinical instability. According to the literature, the sensitivity of the endoscopy increases when both upper endoscopy and colonoscopy are performed, as reported by Mårtensson et al, who notes that the performance of rectosigmoid biopsies has a sensitivity of 85% for the diagnosis of GI GVHD, but when combined with biopsies of the upper GI tract, it reaches 97% (12). Furthermore, 2 pediatric studies comparing the different areas of the GI tract recommend sigmoidoscopy as the first option in children with a clinical suspicion of GI GVHD (13,14). This approach seems to be an attractive strategy, especially for those who are seriously ill, avoiding complete bowel preparation for colonoscopy. In our cohort, bowel preparation was successful only in some patients, with a complete examination being performed in <50%.

The endoscopic appearance of the affected mucosa varies greatly and lacks specificity, with erythema, deep ulcers, and even normal mucosa reported. In our cohort in 20 procedures, we described normal-appearing mucosa with subsequent GVHD confirmed by histology. Additionally, another patient who presented with hematemesis (without diarrhea) also had apoptotic cells in the colon when the endoscopic appearance was normal. Despite this, mucosal exfoliation seems to have a high positive predictive value (15).

The organ with the highest sensitivity for biopsy remains controversial, some recommend gastric biopsies and others recommend rectosigmoid biopsies (16–18). The Lerner classification is the most widely used histopathological scoring system for acute intestinal GVHD based on apoptotic bodies, crypt destruction, and mucosal denudation. According to the National Institute of Health Pathology Working Group, biopsy specimens can be reported as negative for GVHD, possible GVHD, and probable GVHD (19). In our cohort, we observed no direct or proportional relationship between histological classification and endoscopic or colonoscopic classification.

The subgroup of patients with a diagnosis of hepatic GVHD presented with serum transaminase levels over 10× the normal range. However, we did not find high bilirubin values as those described in the literature (20–22). Although liver biopsy is preferred to diagnose organ involvement, it tends to be performed infrequently due to the risk of complications. We estimate that this variable may have delayed the diagnosis in our patients, since 70% were diagnosed after 90 days. In our cohort, the development of hepatitis and cholestasis, in the absence of other causes to explain it, was the main indication for performing a liver biopsy via the transjugular route.

When evaluating which patients presented greater severity of damage, according to the endoscopic and histological classifications, we only found that those with involvement of both organs presented with greater severity according to the colonoscopic classification. This finding would only be relevant if we were able to develop an earlier diagnostic tool for liver involvement, which would allow anticipating serious disease.

Most of our patients required some nutritional support, including parenteral nutrition in the majority of the patients. Although permanent nutritional support is important, the question arises as to whether enteral nutrition would have any role as an immunomodulator, as has been seen in other pathologies, which, in addition to preventing and treating deficits, has a therapeutic effect or improves clinical outcomes (23,24).

We observed a high frequency of invasive infections from coagulase-negative staphylococcus and opportunistic microorganisms (aspergillosis and candida), similar to other reports (25). Other unusual but highly relevant complications relate to endoscopic procedures, such as duodenal hematomas (26) reported to be up to 3.1% by Sahn et al in pediatric patients undergoing endoscopy for suspected GI GVHD (27). In our study, only one patient had a duodenal hematoma and required prolonged enteral rest, without subsequent complications. Another risk secondary to the diagnostic procedure is perforation, as described by Khan et al in their report of 191 children with HSCT undergoing endoscopy; 1 patient died from a perforation of the splenic flexure (28). Fortunately, none of our patients had this complication.

Mortality was similar to that reported in the literature, reaching a rate of 26%, which reinforces the need for prevention, treatment, and early support in these patients.

The retrospective design of our study is the most important limitation, leading to a risk of bias especially in relation to clinical parameters.

GI and liver GVHD are important complications of HSCT, with high morbidity and mortality. Early clinical presentation is common, mainly diarrhea and abdominal pain. Endoscopic involvement is variable and not always related to histology. No single classification system associates severity with worse results in pediatrics; however, GI and liver involvement together could predict a severe target organ involvement. Diagnostic procedures are not exempt from complications, so we recommend performing them in a timely manner and with the corresponding care. More studies should be conducted to identify factors that increase the risk for the development of hepatic and GI GVHD.
